# A longitudinal study of the working relationship and return to work: perceptions by clients and occupational therapists in primary health care

**DOI:** 10.1186/s12875-015-0258-1

**Published:** 2015-04-10

**Authors:** Mona Eklund, Lena-Karin Erlandsson, Birgitta A Wästberg

**Affiliations:** 1 Department of Health Sciences, Lund University, PO Box 157, SE-22100 Lund, Sweden; 2 Skane University Hospital Malmö-Lund, 221 85 Lund, Sweden

**Keywords:** Work rehabilitation, Vocational rehabilitation, Occupational therapy, Outcomes, Helping alliance, Depression, Matched-control design, Social insurance officer

## Abstract

**Background:**

The working relationship between client and therapist can be important to enhance outcomes from vocational rehabilitation for women with stress-related disorders in primary health care. The aim was to investigate the working relationship, as perceived by clients and therapists in the Redesigning Daily Occupations (ReDO™) program, and its relationships to return to work and satisfaction with the rehabilitation. Another aim was to compare the ReDO™ group and a “care-as-usual” (CAU) group regarding perceptions of the working relationship with the social insurance officer.

**Method:**

Forty-two ReDO™ clients and 42 matched controls receiving CAU participated. The study included four measurements (baseline, after 16 weeks rehabilitation and follow-ups after 6 and 12 months). 37 + 37 clients completed. Return to work data was obtained from the Social Insurance Offices (SIO), and the working relationship and client satisfaction were assessed by self-report questionnaires.

**Results:**

The clients rated the working relationship higher than the therapists (mean rating 101.1 vs. 93.9; p < 0.001). The therapists’ rating showed a statistically significant association with return to work at the 12-month follow-up, and the clients’ perceptions were statistically significantly related to how they rated satisfaction with the rehabilitation received. The ReDO™ and the CAU groups did not differ regarding how they rated the relationship with the SIO officer (mean ratings 83.9 vs. 77; p = 0.189). The working relationship with the SIO officer was not related to return to work, but an association (r_s_ = 0.70, p < 0.001) to client satisfaction at 16 weeks appeared in the CAU group alone.

**Conclusion:**

The working relationship as perceived by clients and therapists seemed to be partly separate phenomena, the client perceptions being linked with satisfaction with the rehabilitation and the therapist perceptions with the clients’ return to work. The relationship to the SIO officers was of no importance to return to work but was of some significance for satisfaction with the rehabilitation among the CAU clients. Therapists should strive to improve the relationship with clients to whom they feel the relationship is fragile since that might enhance the chances for those clients to return to work.

**Trial registration:**

Registered at ClinicalTrials.gov (identifier NCT01234961) 2 November 2010.

## Background

Sick leave due to illness is a major problem in Sweden, and a great percentage of sick-listed individuals are on sick leave for more than 60 days. Women constitute about two thirds of those on long-term sick leave (>60 days) [1]. The most common reason for sick leave is mental disorders, which includes depression and stress-related disorders [2]. Different rehabilitation interventions are afforded, aiming at supporting the individual to return to work. In Sweden the local social insurance agencies are responsible for coordinating vocational rehabilitation. For those individuals who are in work the employer has the main responsibility for the vocational rehabilitation [3].

Research has shown that rehabilitation and return to work after a period of sick leave is a complex process and is influenced by different factors such as: appropriateness of interventions [4-7], beliefs in ability to work and that return to work will be feasible [8,9], and access to support and understanding from the authorities and professionals [10-12]. For giving appropriate support the professional has to gain the client’s confidence and establish a trustful relationship.

The importance of a trustful relationship between client and therapist for successful outcomes of interventions is confirmed in studies from a variety of health care areas; in vocational rehabilitation [13,14], mental health care [15] and psychotherapy [16], but also in primary health care [4,17,18]. The relationship is sometimes named differently, although the meaning is about the same: “therapeutic alliance” [17,19], “helping alliance” [15], “working alliance” [13,16], “working relationship” [20], and “therapeutic relationship” [21]. In this study the term “working relationship” will be used.

Researchers state that the working relationship has to be based on trust, perceptions of being understood and listened to and having confidence in the therapist [22,23]. Taylor [24] highlights three major themes of importance for the client-therapist relationship in occupational therapy: collaborative and client-centered approaches, emphasis on caring and empathy, and use of narrative and clinical reasoning. Lustig and co-workers [13] highlight the importance of agreement between the client and the rehabilitation counsellor regarding shared goals for the rehabilitation. On the other hand, a good working relationship facilitates reaching the rehabilitation goals [16].

A fairly recent approach in health promotion and rehabilitation is constituted by activity-based lifestyle interventions, which address a person’s whole pattern of everyday activities, identify aspects that lead to a detrimental pattern, and strengthen the person’s strategies and resources to accomplish change towards a more a satisfying pattern of everyday activities [25,26]. That approach, which uses the group format to reinforce reflection and enhance opportunities for model learning, has also been used in a program aiming at return to work [27]. Vocational rehabilitation is an umbrella term for a variety of methods and it cannot be generalized from the few studies that exist [13,14] that a good working relationship is a pathway towards good vocational outcomes in all programs. More research on the relationship between the working relationship and work outcomes is thus needed to further encircle the importance of the working relationship, and no study seems to have addressed a lifestyle-oriented program for return to work. If the working relationship were found important for vocational outcomes it would be regarded as another intervention ingredient, besides the intervention program per se, that could and should be utilized to promote best possible outcomes.

Researchers have proposed that the working relationship works in mainly two ways; as an active ingredient in itself that contributes directly to an outcome, or as a factor that interacts with and possibly reinforces the effects of the therapy [28]. It is also likely that the working relationship co-varies with factors such as client satisfaction. For theoretical reasons, to help encircle the construct of working relationship vis-à-vis client satisfaction in the vocational rehabilitation context, it would also be interesting to address the relationship between the working relationship as perceived by both clients and staff, and the clients’ satisfaction with the rehabilitation received.

The main aim of this study was to investigate the working relationship, as perceived by clients and occupational therapists in a lifestyle-oriented vocational rehabilitation program, and its relationships to return to work and satisfaction with the rehabilitation. Since the social insurance officer coordinates all rehabilitation measures for people who are off sick, he/she is another person with whom the clients form a working relationship of potential importance for return to work. A second aim was therefore to compare the group receiving the lifestyle-oriented program and a group receiving care as usual (CAU) with respect to how they rated the working relationship they perceived with the social insurance officer. The following research questions guided the study:

- How did clients and occupational therapists in the lifestyle-oriented program rate the working relationship and were there any differences between these ratings?
- How was the working relationship, as perceived by clients and the occupational therapist in the lifestyle-oriented program, related to return to work rate in a short-term and long-term perspective?
- What was the association between the working relationship and the clients’ satisfaction with the rehabilitation?
- Was there any difference in how the clients in the lifestyle-oriented program and the CAU clients rated the relationship with the social insurance officer?

## Methods

This study was part of a project evaluating the effectiveness regarding return to work of a lifestyle-oriented program according to a matched-control design [29]. It was approved by the Regional Ethical Vetting Board at Lund University (Nos. 922/2004 and 149/2007). Participation was based on informed consent and all participants gave their written approval.

### The lifestyle-oriented program

The program in focus was the Redesigning Daily Occupations™ (ReDO™) [27]. Inspired by the Lifestyle Redesign® project [26], it was based on knowledge of women’s everyday occupations [30-32]. The term occupation, as in everyday occupations, is in the present study used to denote all the everyday chores of a person including paid work, household work, hobbies, socializing, etc. The program, described in detail in Erlandsson [27], is a three-part group-based program covering 16 weeks. During the first part (weeks 1 – 6) the focus is on analyzing hindrances for a balanced and health-promoting occupational lifestyle, such as too restricted time for leisure and inequitable distribution between family members with regard to household work. The second part (weeks 6 – 10) concentrates on obstacles for return to work such as unclear expectations and a burdensome work load. The group meets twice a week for 2½ hours per session during these first ten weeks. The group meetings concentrate on problems with everyday occupations and on developing strategies for how to solve them. In between sessions the participants practice the strategies and try to organize their everyday life to a point where they arrive at a satisfying pattern of everyday occupations. The outcomes of those efforts are then discussed and evaluated during the following session. New problems may be identified and, if necessary, strategies and solutions are renegotiated. The third part (weeks 11 – 16) forms a work practice period. If possible the women spend this period at the regular workplace and otherwise at a new workplace of relevance for the goal of returning to work. The group meets twice during this third and last part of the program to provide support and feedback. The intervention aims at raising clients’ awareness of their everyday occupations in terms of what they do, how they do it and how they perceive it [27].

The group leaders were two occupational therapists who had received specific training in the ReDO™ method. The program was carried out in primary health care in the south of Sweden during 2007–2010. Being a woman, having an employment and currently being on sick leave due to stress-related disorders were the eligibility criteria. The primary outcome of the ReDO™ was return to work, and the intervention was shown to be successful in that respect compared to CAU [29]. It was also effective with regard to decreased degree of sick leave, improved self-esteem [29], and increased quality of life [33] compared to CAU, further characterized below.

### The CAU interventions

CAU included different rehabilitation interventions, the main alternative being support from the social insurance officer and the employer in accordance with Swedish routines. The interventions that formed CAU varied from regular follow-up meetings with the social insurance officer to structured vocational rehabilitation. Other examples were mindfulness training and physiotherapy. About half of the CAU clients received some kind of additional rehabilitation besides the meetings with the social insurance officer.

### Power calculation

The ReDO™ study was the first if its kind – using a lifestyle-based intervention to enhance return to work – and there was no relevant previous study on which we could base the power calculation. This made us reason in terms of effect sizes in general, as described by Altman [34], and the ambition was to detect an effect size of 0.6 between the groups, which corresponds to a moderately strong effect [35]. An effect of that size was deemed large enough to be clinically interesting and significant [36]. Effect size was in the present study defined as the mean of the ReDO™ group minus the mean of the CAU group, divided by the pooled standard deviation. According to a power analysis based on effect sizes, 40 women in each group were required to detect that effect size with 80% power at p < 0.05 [34].

### Participants

Women on long-term sick leave due to stress related ill-health were selected by staff at the Social Insurance Office, who assessed whether the ReDO™ would be an appropriate intervention for them. The criterion of stress-related ill-health was determined on the basis of the general practitioner’s certificate, which was mandatory for receiving sickness benefit from the Social Insurance Office. The doctor’s certificate included one or several ICD-10 diagnoses. Diagnosis was not the only factor that was weighed into the assessment that lay behind the certificate, however, the woman’s anamnesis and total life situation were also important. As a result the main diagnoses were in most cases depression (F32) or stress/exhaustion (F43), but in a few cases the main diagnosis was a physical disorder in terms of cervical disc disorder (M50).

The ReDO™ program was a new intervention, not known to potential clients, but all women who were offered the intervention accepted the invitation. They also agreed to take part in the study. A total of 42 women entered the program and were distributed into ten groups. All of them agreed to participate in the research project, and 37 clients completed the data collection for this study. The CAU group was selected from the Social Insurance Office register by a matching procedure. Women receiving CAU and who matched the ReDO™ women on age (+/− 5 years), education level, being married or not, number of children and time on sick leave were selected. Of the 42 women who were chosen and agreed to participate (75% of those who were approached), 37 completed the data collection on which this study was based. When a CAU woman declined, another who also matched the ReDO™ woman was selected. She thus had equivalent age, family situation, profession, etc., in comparison with the woman who declined. Data that would enable a comparison between those who declined and the final CAU sample was not available. Socio-demographic characteristics of those 37 + 37 clients who completed the data collection are presented in Table 1. The two groups were comparable on all variables presented in the tables. No statistically significant difference was found. Nor were there any statistically significant differences between the 74 participants and those 10 who did not complete all instruments for the data collection.

**Table 1:** **Characteristics of the participants**

	*The ReDO™ group N = 37*	*The CAU group N = 37*
Age, mean (SD)	45 (9.9)	46.7 (8.7)
Married, n (%)	27 (73)	24 (65)
Number of children	2.4	2
College/University education; n (%)	14 (38)	17 (46)
Diagnosis; n (%)		
Depression; F32	21 (57)	17 (46)
Stress/Exhaustion; F43	15 (41)	17 (46)
Physical main diagnosis; M50	1 (3)	3 (8)
Months on sick leave, mean (SD)	9.1 (9)	10.6 (10.7)

### Data collection

The Social Insurance Offices’ registers were consulted to obtain information regarding the clients’ diagnoses and return to work. The percentage of the woman’s ordinary working hours was used as an estimate of degree of return to work. For example, a woman who at a given point worked 30 hours per week, but was normally working 40 hours per week, was seen as having returned to work at 75%.

A background questionnaire was developed for the project and addressed socio-demographic data. The below instruments were used to gather further data.

### Working relationship

The instrument used to assess the working relationship was the Working relationship Questionnaire II (HAqII) [37]. It is based on self-report and is composed of 19 items such as “I feel the therapists want me to achieve my goals” and “A good relationship has formed with my therapists”. The HAqII was developed to assess the relationship with one single therapist, but it has also been successfully used to address the working relationship perceived in relation to a staff group as a whole [15,38]. This is how the HAqII was used in the present study. The response format is a Likert-type scale with six alternatives ranging from 1 (I strongly believe it is not true) to 6 (I strongly believe it is true). Reversed scores are applied for five negatively worded items. A higher score thus indicates a better working relationship and the maximum obtainable score is 114. The HAqII has shown excellent internal consistency and test-retest reliability and good convergent validity [37]. The therapists filled out a staff version with analogue wording. Both the client version (alpha = 0.91) and the staff version (alpha = 0.95) showed excellent internal consistency and all corrected item-total correlations were larger than 0.20, as proposed in the literature on psychometrics [39].

### Client satisfaction

The Client Satisfaction Scale, shown to have good psychometric properties [40,41], was used as a self-report of satisfaction with the rehabilitation received. It has eight items consisting of statements that are rated on a four-point scale from very dissatisfied (=1) to very satisfied (4), giving a highest possible score of 32. The Cronbach’s alpha coefficient for the current sample was excellent at 0.94.

### Procedures

Research assistants collected the data at individual appointments with each woman. The appointments were in the premises where the ReDO™ women received the intervention. For the CAU women the appointments were either in the woman’s home or in a neutral place, such as a café, at the woman’s discretion. For both groups, the appointments took place in a comfortable environment. The working relationship was rated after the first 10 weeks of the 16-week rehabilitation, in order for a working relationship to have stabilized. The client rated the working relationship as perceived with the occupational therapists as a team and each occupational therapist rated the individual relationship they felt they had with each client. There were thus two occupational therapist ratings for each client, but in analogy with the fact that each client gave one joint rating of the working relationship with the two therapists the therapist ratings of the working relationship were combined to a mean rating for each client. Client satisfaction was assessed after both 10 and 16 weeks. Return to work data were compiled at completion of the 16-week rehabilitation and at follow-ups 6 and 12 months after completion. The data for the CAU were collected on corresponding occasions. For natural reasons the only working relationship the CAU rated was that with the social insurance officer.

### Data analysis

The instruments used to assess the working relationship and client satisfaction formed ordinal scales and non-parametric statistics were thus used. Group comparisons were made by means of Mann–Whitney tests (for independent samples) or Wilcoxon’s test (for related samples). Spearman’s rank correlation test was used to analyze relationships between variables.

## Results

The ReDO™ clients’ mean rating (SD) of the working relationship they perceived with the occupational therapists was 104.1 (8.1). The corresponding mean rating from the occupational therapists was 93.9 (8.2). The higher rating from the clients was statistically significant as compared with occupational therapist rating (Z = −4.24, p < 0.001).

The relationships between ratings of the working relationship and degree of return to work among the ReDO™ clients are shown in Table 2. The client rating was not associated with return to work at any measurement. The therapists’ rating showed a statistically significant association with return to work at the 12-month follow-up, but not with the return to work situation at completion of the intervention or at the 6-month follow-up.

**Table 2:** **Relationships between the working relationship, degree of return to work and client satisfaction in the ReDO™ group**

	*Client rating of the working relationship (Mean = 104, SD = 8)*	*Occupational therapist rating of the working relationship (Mean = 94, SD = 8)*
Degree of return to work (% of previous working hours) at completion of the intervention (Mean = 20, SD = 32)	ns.	ns.
Return to work (% of previous working hours) at 6 months (Mean = 51, SD = 41)	ns.	ns.
Return to work (% of previous working hours) at 12 months (Mean = 69, SD = 39)	ns.	r_s_ = 0.53, p < 0.001
Client satisfaction at 10 weeks (Mean = 26, SD = 5)	r_s_ = 0.36, p = 0.034	ns.
Client satisfaction at 16 weeks (Mean = 27, SD = 5)	r_s_ = 0.38, p = 0.025	ns.

Also shown in Table 2 are the associations between the working relationship and client satisfaction for the ReDO™ group. The clients’ perceptions of the working relationship were statistically significantly related to how they rated their satisfaction with the rehabilitation received, after both 10 weeks and 16 weeks of rehabilitation. No statistically significant associations between the therapists’ rating of the working relationship and client satisfaction were found.

Regarding the working relationship to the responsible social insurance officer, the mean (SD) rating from the ReDO™ clients’ was 83.9 (18.8) and that from the CAU clients was 77 (22.7). No group difference was indicated (Z = −1.314, p = 0.189). When the groups were combined there was no relationship between the clients’ rating of the working relationship with the social insurance officer and return to work or satisfaction with the rehabilitation received at any time, p-values ranging between 0.199 and 0.904. When the correlational analyses were made separately for the two groups, however, a statistically significant relationship (r_s_ = 0.70, p < 0.001) was found between the working relationship rating and client satisfaction at 16 weeks in the CAU group. All other relationships remained non-significant.

## Discussion

The ReDO™ clients’ gave high ratings of the working relationship. A reason for that might be that the occupational therapists used a collaborative and client-centered approach. They supported the clients in working with their own occupational situation towards clear goals, as indicated in an interview study that included a purposeful sample of ReDO™ participants [42]. The importance of a collaborative and client-centered approach for enhancing the working relationship in rehabilitation has been highlighted by several researchers [4,24,43,44].

That the working relationship, as assessed during the program, was associated with work outcomes and client satisfaction to some extent must be seen as the core findings of this study. Starting with the associations between the working relationship as rated after 10 weeks rehabilitation and client satisfaction, it was obvious that these were partly overlapping phenomena on the clients’ behalf. This was found for both client groups and is supported by a literature review [19] pointing to associations between the working relationship and perceived quality of care and client satisfaction. It may be that the involved factors mutually reinforce each other, thus that the clients’ perceptions of a good relationship increase their satisfaction with the rehabilitation and vice versa. When addressing the non-significant relationship between the clients’ rating of the working relationship and return to work it is important to bear in mind that although the ReDO™ intervention is a rehabilitation program aimed at return to work, the content of the program targets everyday life as a whole. Altering some aspects of everyday life in general was for many clients a prerequisite for return to work [42]. Thus it might be that the clients’ ratings of the working relationship were influenced by a broad range of factors, more linked with the program as a whole (and thus client satisfaction) than with return to work.

The working relationship as rated by the occupational therapists was not at all related with client satisfaction. On the other hand the therapists’ ratings were associated with return to work 12 months after completed ReDO™ program. The therapists’ perceptions of the working relationship thus seemed to reflect their judgment of whether the client was developing towards enhanced work ability, which seems a reasonable conclusion in the vocational rehabilitation context. The lack of a statistically significant correlation with return to work in the short-term perspective, after completed rehabilitation and at the six-month follow-up, must be seen against the fact that the clients were then in transition from having been on sick leave and that this process went faster for some clients and slower for others. The ReDO™ clients’ rating of the working relationship they perceived with the occupational therapists was not associated with return to work, which indicates that the relationship as rated by clients and therapists were at least partly different phenomena.

Furthermore the ReDO™ clients rated the working relationship higher than the therapists did which is in line with previous research within areas such as occupational therapy [45], general psychiatric care [15,38] and psychotherapy [20]. This further underscores that clients and therapists may partly have rated varying phenomena and referred to different aspects of the relationship. As discussed above, the clients might have had the program as a whole in mind when they rated the working relationship whereas the therapists might have focused on how well the relationship worked with respect to return to work. Another explanation to the fact that clients often rate the relationship higher than the therapists may lie in social desirability, as proposed in a review by Tryon and co-workers [20]. They discussed that the wish to stand out in a favorable way could be stronger among clients than among therapists and that the working relationship might be perceived as more important by the clients than by the therapists.

It was obvious that the working relationship the ReDO™ and CAU clients had developed with the social insurance officer, as perceived by the clients themselves, was of no importance for the degree of return to work in the short-term or long-term perspective. Nor was it related with satisfaction with the rehabilitation for the ReDO™ clients. A strong relationship [36] appeared however between the CAU clients’ rating of the working relationship with the social insurance officer and their satisfaction with the rehabilitation received. An interpretation of these findings is that the relationship to the social insurance officers was of subordinate significance to the ReDO™ clients, for whom the relationship with the occupational therapists providing the intervention was probably of more importance. For the CAU clients, however, the social insurance officers were sometimes the only support at hand. Still, since the social insurance officers are part of the support system for all persons on sick leave and thus important to the clients who depend on these services, one could expect this relationship to be of significance also for the ReDO™ clients. That was not indicated by the result but it should not be concluded from this single study that the relationship between client and the social insurance officer is unimportant for successful vocational rehabilitation.

The fact that there were no associations between the working relationship and return to work from the clients’ perspective deserves some further attention. Other research has demonstrated such associations in the vocational rehabilitation context [13]. When considering the literature on associations between client perceptions of the working relationship and outcomes in general, the result picture is also not unanimous. For example, type of therapy has shown to impact on that association. Arnow et al. [46] found that a directive form of therapy for depression entailed a closer link between the working relationship and outcome than did a non-directive therapy, although they had hypothesized the opposite. It is possible that complex interventions such as vocational rehabilitation, which is influenced by many factors on the societal and personal levels [4,5,8], make it more difficult to discern an association between the client’s view of the working relationship and return to work. Studying the impact of the working relationship in the vocational rehabilitation context is a fairly new line of research, and future studies will reveal whether or not a consistent pattern of associations can be identified.

### Implications for how to enhance the working relationship

The findings indicate that the working relationship should be considered in vocational rehabilitation and all professionals involved should strive to enhance that relationship. But how can that be done? Cabaniss and colleagues [47] proposed some strategies for how to promote the working relationship between the therapist and the client, which may be transferred to the vocational rehabilitation context. The professionals should show interest in the client and in what is troubling him or her. This also means the professionals should show attention and give the client time to tell his or her story, as also emphasized by Taylor [24]. Such strategies are particularly important in the initial phase of vocational rehabilitation, for example the social insurance officer’s or the therapist’s first meetings with the client. The professionals should also convey that they understand something about the client’s problem, including that they have a sense of the severity of the client’s problem and how difficult or painful it is to him or her [24,47]. In order to develop a sense of trust, the client must be able to feel that the social insurance officer or the therapist is both competent and understanding. Research has also found that clients prefer facts and clear explanations, but do not want professionals to become too emotional [48]. Cabaniss et al. [47] advised that therapists can show some emotions to express their empathy, but they should not refer to their own situation or problems [48]. These are measures to enhance the working relationship, which according to some findings from the present study, and also previous research [13], may increase the chances for clients’ return to work.

### Methodological considerations

It was not possible to obtain the social insurance officers’ ratings of the working relationship in the present study. Investigating the working relationship from their perspective might have revealed information that could benefit the rehabilitation process. The fact that this study lacks their perspective limits the result picture and future research should include that perspective as well. Another methodological issue concerns the time point for the measurement of the working relationship. With the argument that a working relationship will take time to develop, the measurement for the present study was made after about two thirds of the rehabilitation period. Others have argued that the early relationship is of vital interest [46,49], and also that repeated measurements of the working relationship are preferred [50]. It is possible that other measurement points would have yielded other findings, such as an association between the working alliance and return to work also from the clients’ perspective. Additionally, the number of participants was quite small, and the drop-outs entailed that the study was somewhat under-powered in relation to the power analysis. True associations may therefore have gone undetected because of type-II errors. It should also be acknowledged that all findings, particularly those that pertain to the working relationship to the social insurance officer, may not be generalizable to societies with social insurance systems that are basically different from that of Sweden.

## Conclusions

The findings indicated that the working relationship as perceived by clients and occupational therapists were partly separate phenomena. The clients’ perceptions were closely related with their satisfaction with the rehabilitation received, whereas the therapists’ perceptions were associated with the degree to which the clients returned to work. The working relationship to the social insurance officers was of subordinate importance to both satisfaction and return to work for the ReDO™ clients, but it was of significance for the CAU clients, for some of whom the social insurance officer was the only rehabilitation contact during sick leave.

The working relationship should thus be considered in vocational rehabilitation and all professionals involved should strive to enhance that relationship. In particular, those who deliver the services procured by the Social Insurance Offices must be attentive in this respect. This study also suggests that the professionals can take measures to improve the relationship with clients to whom they feel the relationship is fragile and thus enhance the chances for those clients to return to work.

## Abbreviations

CAU: Care as usual
GAF: Global Assessment of Functioning
HAqII: Working relationship questionnaire II
ReDO™: Redesigning Daily Occupations™
SIO: Social Insurance Office
